# Prognostic value of the C-reactive protein to albumin ratio in patients undergoing primary percutaneous coronary intervention for ST-segment elevation myocardial infarction

**DOI:** 10.3906/sag-2003-188

**Published:** 2021-06-28

**Authors:** Özgür SÖĞÜT, Tarık AKDEMİR, Mehmet Mustafa CAN

**Affiliations:** 1 Department of Emergency Medicine, Haseki Training and Research Hospital, University of Health Sciences, Istanbul Turkey; 2 Department of Cardiology, Haseki Training and Research Hospital, University of Health Sciences, Istanbul Turkey

**Keywords:** CRP/albumin ratio, infarction localization, prognosis, ST-elevation myocardial infarction, vascular lesion number

## Abstract

**Background/aim:**

This study investigated whether baseline serum level of C-reactive protein (CRP)/albumin ratio is associated with infarct localization, number of vascular lesions, and in-hospital mortality in patients undergoing primary percutaneous coronary intervention (PCI) for acute ST elevation myocardial infarction (STEMI).

**Methods:**

The study population consisted of 116 patients diagnosed with STEMI. The CRP/albumin ratio at first admission, cardiac troponin-I (cTnI), PCI results, and clinical outcomes were recorded.

**Results:**

The mean CRP/albumin ratio, cTnI level, and mean number of vascular lesions were significantly higher in non-survivors than in survivors (p = 0.006, p = 0.004, and p = 0.007, respectively). Multivariate logistic regression analysis demonstrated that the CRP/albumin ratio and number of coronary artery lesions were independent predictors of mortality in STEMI patients. According to these analyses, the presence of ≥ 2 vessel lesions was the most important predictor of mortality, with an odds ratio of 2.009 (95% confidence interval: 1.191–3.387, p = 0.009).

**Conclusion:**

This study demonstrates the potential utility of the CRP/albumin ratio for predicting the clinical outcome of patients with STEMI. In addition, the presence of ≥ 2 vascular lesions contributed to a 2-fold increase in mortality rate in STEMI patients.

## 1. Introduction

Cardiovascular diseases, including coronary artery disease (CAD), stroke, heart failure, peripheral artery disease, carotid artery diseases, and aortoiliac diseases, are the most important causes of mortality and morbidity worldwide [1]. CAD occurs as a result of narrowing of the blood vessels by atherosclerosis, which in turn causes a reduction in the amount of oxygen delivered to the cardiac muscles, thus leading to unstable angina, myocardial infarction (MI), and heart failure [2]. 

The risk factors of CAD are well known and include age, male sex, family history, smoking, hypertension, diabetes, obesity, high total cholesterol level, high-density lipoprotein cholesterol deficiency, excess of low-density lipoprotein cholesterol, triglyceride level, a sedentary lifestyle, and an increased waist circumference [2,3]. 

Atherosclerosis is an inflammatory disease that results in CAD. Inflammation in the endothelium of the coronary arteries is associated with increases in acute phase proteins and cytokines. Focal and systemic inflammation play key roles in the destabilization and rupture of atherosclerotic plaque [4]. Consequently, inflammatory biomarkers are being intensively studied for their ability to determine the severity and prognosis of patients with CAD [5,6]. One such biomarker is C-reactive protein (CRP), a prototype marker of the inflammatory process that contributes to CAD and is of interest as a biomarker of the disease [6,7].

Epidemiological studies have shown a relationship between hypoalbuminemia and the development of CAD [8,9]. In patients with CAD, hypoalbuminemia has also been identified as a risk factor for the development of new MI but there is no evidence of the prognostic value of serum albumin levels in ST elevation myocardial infarction (STEMI) patients [10,11]. Thus, a number of studies have investigated the relationship between the serum CRP/albumin ratio and inflammatory diseases such as sepsis, cancer, acute pancreatitis, ulcerative colitis, hepatitis B, and CAD [5–14]. 

The ratio of serum levels of CRP to albumin has been demonstrated to be a more accurate indicator than albumin and CRP levels alone for determining the prognosis of patients with various inflammatory diseases such as sepsis, cancer, acute pancreatitis, ulcerative colitis, and hepatitis B [12–20]. As atherosclerosis, and therefore CAD, is an inflammatory process, the serum CRP level may correlate with the severity of atherosclerosis and the size of the atherosclerotic plaque [6,21].

In recent studies, CRP/albumin ratio, a novel indicator of the inflammatory response, was shown to be of moderate value as a marker of CAD and valuable in predicting the development of stent restenosis and disease prognosis following treatment with percutaneous coronary intervention (PCI) [19,22,23]. However, the relationship between the serum CRP/albumin ratio and clinical outcome in patients undergoing PCI after acute MI is not yet clear. 

Therefore, the present study investigated whether the baseline CRP/albumin ratio is associated with infarct localization, number of vascular lesions, and in-hospital mortality in patients undergoing PCI following a diagnosis of acute STEMI.

## 2. Materials and methods

### 2.1. Study design and setting

This study was conducted in accordance with the 1989 Declaration of Helsinki and was approved by the Ethics Committee of the Faculty of Medicine, University of Health Sciences, İstanbul Research and Training Hospital (ethical approval number 1453). The study population in this prospective study consisted of 116 consecutively enrolled adult patients admitted to the emergency department (ED) of our tertiary care university hospital between October 2018 and January 2019, where they were diagnosed with STEMI. After the patient’s vital functions had been monitored, written informed consent was obtained directly from the patient or from his or her authorized representative. 

### 2.2. Selection of participants

Patients included in the study were those admitted to the ED for chest pain or ischemia equivalent (especially elderly, diabetic, and female patients, with symptoms such as dyspnea, fatigue, and dizziness). All 116 patients were subsequently diagnosed with ECG-confirmed acute STEMI for which they underwent PCI and were then admitted to the coronary intensive care unit (ICU). Age, sex, smoking history, vital signs, electrocardiography (ECG), symptoms, symptom duration, CRP/albumin ratio at first admission, coronary angiography, and PCI results (number of vascular lesions) were determined. The clinical outcome (in-hospital mortality) was recorded in a pre-formatted case data form. Blood samples were taken from the patients before PCI and used to measure CRP, albumin, and cardiac troponin-I (cTnI; reference weight 0.02–0.06 ng/mL) levels. The relationships between the CRP/albumin ratios as determined at first admission and infarct localization, and number of vascular lesions were analyzed. In addition, patients who were hospitalized and then died in the hospital vs. those who survived and were discharged were compared in terms of their CRP/albumin ratio at first admission. 

The inclusion criteria were patients with chest pain and ischemia equivalent symptoms, age 18 years and over, with an ECG-confirmed diagnosis of STEMI treated with PCI, and oral or written consent obtained from the patient or his/her guardian. Exclusion criteria were individuals under 18 years of age, patients with cardiac arrest before PCI, patients with non-STEMI; patients/guardians who did not provide oral or written consent; patients who within the last 1 week took medications that may affect the CRP level (antiinflammatory, antibiotic, statins, etc.); patients with accompanying systemic infection.

### 2.3. Statistical analysis

The required sample size was calculated by power analysis before data collection. It was estimated that at least 111 patients would be required to detect significant differences among the numerical variables with a power of 95% and an alpha error of 5%. All analyses were conducted using SPSS statistical software (version 15.0 for Windows; SPSS, IBM Corp., Armonk, NY). Numerical data (e.g., CRP/albumin, neutrophil/lymphocyte, cTnI level, number of vascular lesions, and symptom duration) are expressed as means ± standard deviations (SDs) and minimum and maximum (min–max) values, and categorical variables (sex and age) are expressed as numbers (n) and percentages (%).

The distribution of the included patients’ data was examined for normality. Therefore, intergroup comparisons (survivors vs. nonsurvivors) were conducted using the independent-samples *t* test and chi-square test for normally distributed data and Mann–Whitney U-test for nonnormally distributed data. Intragroup comparisons (STEMI groups) were made using the Kruskal–Wallis test. To normalize the distributions, extreme values were ignored, but that did not eliminate the distortions of the variables. Spearman’s rank correlation test was used to evaluate the correlation between the CRP/albumin ratio and the number of vascular lesions. Multivariate logistic regression analysis was performed to identify significant predictors of mortality, including serum levels of CRP/albumin, hs-cTnI, and number of vascular lesions. Confidence intervals were given at the 95% level (95% CI) and p < 0.05 was taken to indicate statistical significance.

## 3. Results

The study population consisted of 100 male (86.2%) and 16 female (13.8%) patients with STEMI. Their mean age was 56.47 ± 11.42 years (range: 28–86 years). There were no statistically significant differences between survivors and non-survivors in terms of age, sex, and symptom duration parameters (p = 0.125, p = 0.700, and p = 0.569, respectively; Table 1). Likewise, there were no statistically significant differences between survivors and non-survivors in terms of the mean albumin levels (4.40 ± 3.01 vs. 3.86 ± 0.44, respectively, p = 0.457) or CRP (12.75 ± 18.31 vs. 25.34 ± 33.66, respectively, p = 0.139).

**Table 1:** Comparison of demographic, clinical, laboratory and coronary angiographic characteristics in surviving and non-surviving patients.

Characteristic		Survivors (n = 98)		Non-survivors (n = 18)		
		Mean ± SD	Min–max	Mean ± SD	Min–max	p-value*
Age		55.78 ± 10.95	28–86	60.28 ± 13.42	40–85	0.125
Sex (n, %)	Male	85 (86.7%)		15 (83.3%)		0.700
	Female	13 (13.3%)		3 (16.7%)		
CRP/albumin (mg/L/g/dL)		3.18 ± 4.99	0.11–25.88	6.41 ± 8.01	0.21–30.82	0.006
cTnI (ng/mL)Number of vascular lesionsSymptom duration (h)		3.26 ± 10.111.61 ± 0.858.67 ± 15.61	0.01–68.300–40.50–72	4.94 ± 11.022.39 ± 1.1916.50 ± 23.40	0.01–46.821–40–72	0.0040.0070.569

Note: Data are expressed as numbers, percentages, mean ± standard deviation (SD), and minimum and maximum (Min–max) values. *Intergroup comparisons (survivors vs. non-survivors) were made using an independent sample t-test as well as chi-squared and Mann–Whitney U tests as appropriate.Abbreviations:

**Figure 1:**
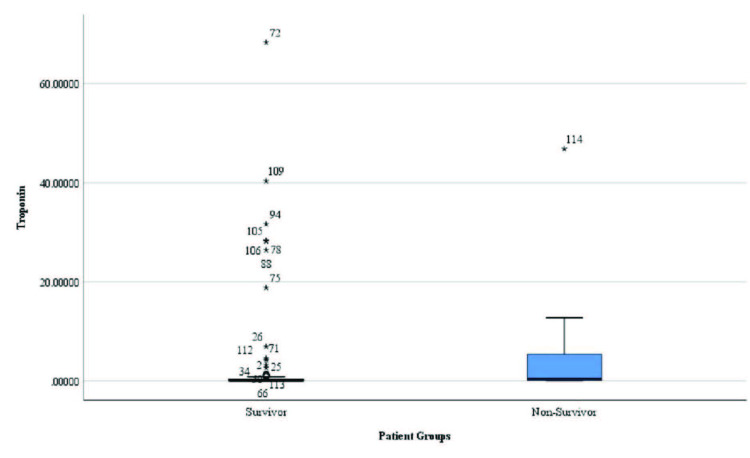
Distribution of cardiac troponin-I levels in survivors and non-survivors.

**Figure 2:**
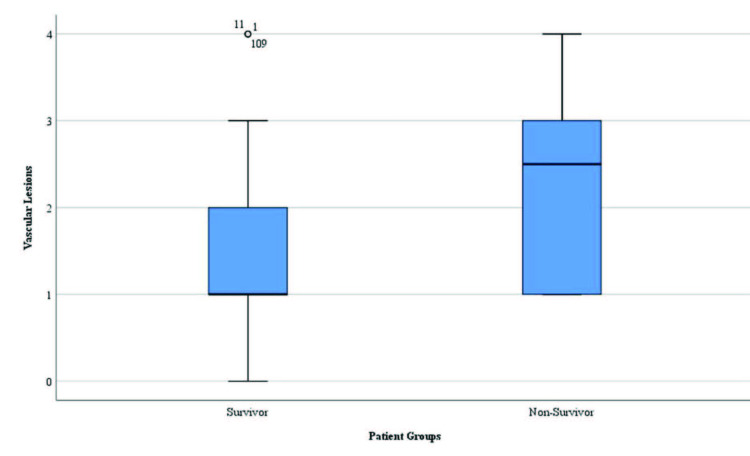
Distribution of the number of vascular lesions in survivors and non-survivors.

**Figure 3:**
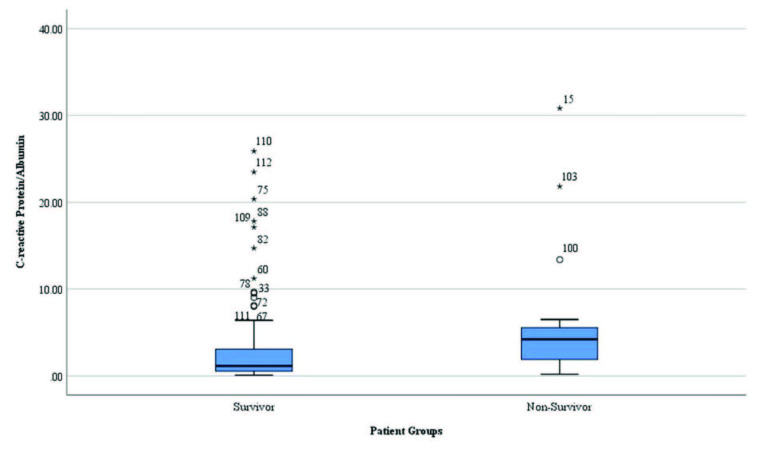
Distribution of the C-reactive protein/albumin ratio in survivors and non-survivors.

However, the mean CRP/albumin ratio, cTnI level, and mean number of vascular lesions were significantly higher in non-survivors than in survivors (p = 0.006, p = 0.004, and p = 0.007, respectively; Table 1 and Figures 1–3).

In multivariate logistic regression analyses, the CRP/albumin ratio and number of coronary artery lesions remained independent predictors of mortality in patients diagnosed with STEMI. The number of vascular lesions was the most important predictor of mortality, with an odds ratio of 2.009 (95% CI: 1.191–3.387, p = 0.009; Table 2). In fact, the presence of ≥ 2 vascular lesions increased the risk of mortality in STEMI patients by 2-fold.

**Table 2:** Independent predictors of mortality in patients with STEMI identified in multivariate logistic regression analysis.

Predictor	Beta	OR (95% CI)	SD	p-value
CRP/albumin (mg/L/g/dL)	0.166	1.249 (0.966–2.139)	0.153	0.038
cTnI (ng/mL)	0.008	1.008 (0.953–1.066)	0.029	0.786
Number of vasculer lesions	0.697	2.009 (1.191–3.387)	0.266	0.009

Abbreviations: STEMI, ST elevation myocardial infarction; CRP, C-reactive protein; cTnI, cardiac troponin-I; Beta, standardized regression coefficient; OR, odds ratio; CI, confidence interval; SD, standard deviation; P, statistical significance.

The patients were also classified according to the ECG-based MI localization as follows: group I: inferior STEMI, group II: anterior STEMI, and group III: other (lateral, right, posterior, etc.) STEMI. While there was no statistically significant difference between the groups in terms of mean CRP/albumin ratio (p = 0.119 and Table 2), mean serum cTnI levels were significantly higher in group II (anterior STEMI) than in group I (inferior STEMI) patients (4.56 ± 10.56 and 0.63 ± 3.03, respectively; p = 0.039; Table 3). The difference in the serum cTnI level between the other groups was not statistically significant ( *p* > 0.05; Table 3).

**Table 3:** Comparison of the serum CRP/albumin ratio and cTnI level among STEMI patients grouped according to the ECG-based MI localization.

Parameter	Group I(n = 38)	Group II(n = 57)	Group III(n = 19)	p-value
	Mean ± SD	Mean ± SD	Mean ± SD	
CRP/albumin (mg/L/g/dL)	3.16 ± 5.12	3.63 ± 5.47	5.14 ± 7.31	0.539
cTnI (ng/mL)	0.63 ± 3.03a*	4.56 ± 10.56b**	6.08 ± 16.55c***	0.039

aGroup 1 vs. group 2: *p = 0.032bGroup 2 vs. group 3: **p > 0.976cGroup 3 vs. group 1: ***p > 0.437

The patients were also grouped according to the number of vascular lesions following PCI. Group 1 consisted of those with 0 or 1 vascular lesions, and group 2 consisted of those with ≥ 2 lesions. The two groups did not significantly differ in their symptom duration, CRP/albumin ratio, and cTnI levels (p = 0.305, p = 0.614, and p = 0.464, respectively; Table 4). By contrast, the mean age of group 2 patients was significantly higher than that of group 1 patients (p = 0.031; Table 4). Finally, there was no significant relationship between the CRP/albumin ratio and the number of vascular lesions (Spearman’s correlation coefficient, ρ = 0.121, p = 0.199).

**Table 4:** Comparison of patient age, symptom duration, serum CRP/albumin ratio, and serum cTnI levels among STEMI patients grouped according to the number of vascular lesions.

Parameter	Group 1(n = 61)		Group 2(n = 55)		
	Mean ± SD	Min–max	Mean ± SD	Min–max	p-value*
Age	54.31 ± 11.33	29–86	58.87 ± 11.14	28–79	0.031
CRP/albumin (mg/L/g/dL)	2.97 ± 4.66	0.13–30.82	4.49 ± 6.55	0.11–25.88	0.614
cTnI (ng/L)Symptom duration (h)	4.33 ± 12.478.52 ± 15.46	0.01–68.300.00–72.00	2.61 ± 6.9611.04 ± 18.43	0.01–40.350.50–72.00	0.4640.305

Note: Data are reported as number, mean ± standard deviation (SD), minimum and maximum min–max). *An independent sample t-test was used to compare the sex distribution of the two groups, and the Mann–Whitney U test to compare symptom duration, serum CRP/albumin ratio, and serum hs-cTnI levels between groups.Group 1, 0–1 vascular lesions; group 2, ≥ 2 vascular lesions. Abbreviations

## 4. Discussion

This is the first clinical study to evaluate the relationships between serum CRP/albumin levels and infarct localization, number of vascular lesions, and in-hospital mortality in the early period in adult patients diagnosed in the ED with acute STEMI and treated with PCI.

Acute coronary syndrome is the most important potentially fatal disease and its occurrence does not differ between males and females [24]. Complications and death due to STEMI have decreased significantly in recent years, due to current and evidence-based treatment modalities [25]. Atherosclerosis-induced CAD is an inflammatory process, and the serum CRP level, as a marker of inflammation, correlates with disease severity and plaque width [19]. Recent studies have shown that the CRP/albumin ratio is related to the clinical outcome in various diseases, and may be a prognostic indicator of CAD [12–15,19,20]. 

The use of the CRP level to predict coronary events has been examined [6]. Wada et al. [19] evaluated 2164 patients diagnosed with CAD who underwent PCI. Based on an average follow-up of 7.5 years, low serum albumin levels together with high CRP levels were shown to be associated with long-term adverse cardiac events (all-cause death and nonfatal myocardial infarction). In addition, low serum albumin and high CRP levels had a synergistic effect on the risk of developing long-term adverse cardiac events in patients undergoing PCI.

In the present study, mean CRP/albumin levels were significantly higher in patients who eventually died than in those who survived to hospital discharge. This result is in accordance with the study of Wada et al. In addition, serum cTnI levels and the number of vascular lesions were significantly higher in non-survivors. In contrast, there were no significant differences in the age, sex distribution, and symptom duration of survivors vs. non-survivors. In our study of STEMI patients who underwent PCI, we investigated the predictive value of the serum CRP/albumin ratio as measured at first admission, which increased the utility of our findings compared to those of Wada et al *.* Thus, the early determination of cTnI levels and serum CRP/albumin levels may serve as a new biomarker to predict a poor clinical outcome in patients with STEMI. In our study, multivariate logistic regression analysis showed that ≥ 2 coronary artery lesions doubled the risk of death, and the CRP/albumin ratio was another independent predictor of mortality in patients diagnosed with STEMI. 

A previous study showed that CRP is not an appropriate diagnostic marker of acute MI in patients presenting to the ED with chest pain. However, serum CRP values more than one-third higher than the upper limit are associated with increased in-hospital adverse cardiac events in these patients [26]. Similarly, in the present study, a comparison of survivors and non-survivors (in-hospital mortality) showed that a high CRP/albumin ratio, measured at first admission associated with in-hospital mortality in the short term. A recent study showed that decreased albumin level is an independent predictor of in-hospital mortality in patients with ACS [27]. Another recent study demonstrated that an increased CRP/albumin ratio can predict coronary microvascular dysfunction better than CRP or albumin alone in patients with celiac disease [28]. Consistent with those findings, we found that the mean CRP/albumin ratio was significantly higher in survivors than non-survivors, despite no significant differences in the mean albumin levels or CRP alone between the two groups. In addition, the increased CRP/albumin ratio remained an independent predictor of in-hospital mortality in patients diagnosed with STEMI.

It has been implicated that the total quantity of cardiac enzyme released into plasma correlates with the infarct size [29]. Previous studies reported that patients with the maximum cTnI level more likely had anterior wall MI with consequent large infarct size [30,31]. In the present study, when STEMI patients were examined according to MI localization (group I: inferior MI, group II: anterior MI, and group III: lateral, right, posterior, etc.), there was no statistically significant difference between the groups in terms of their mean serum CRP/albumin ratio. However, the mean serum cTnI level was significantly higher in group II (anterior MI) than in group I (inferior MI) patients.

Increased age is one of the most important risk factors of CAD, increasing its incidence and prevalence [32,33]. In the present study, among patients evaluated according to the number of vascular lesions after PCI (group 1 with 0–1 and group 2 with ≥ 2 vascular lesions), patients in group 2 were significantly older than those in group 1, whereas there were no statistically significant differences between the groups in terms of symptom duration, serum CRP/albumin ratio, or cTnI level. However, we did find that the number of vascular lesions increased with age and that the presence of ≥ 2 vascular lesions was the most important predictor of mortality in patients with STEMI. These findings imply that advanced age is the most important risk factor for CAD.

This study had some limitations, the most important of which were the small sample size and single-center design. In addition, we were able to obtain data containing only one-time point measurement for cTnI and inflammation parameter, such as CRP/albumin ratio, for each patient. Blood samples were taken upon admission of the subjects to the ED. These issues should be addressed in future studies involving patients with STEMI.

In conclusion, this study demonstrates the value of the CRP/albumin ratio, cTnI level, and number of affected coronary vessels in predicting the short-term clinical outcome of STEMI patients undergoing primary PCI. We also revealed that the number of vascular lesions in these patients increased with age, and in multivariate logistic regression analyses age was an independent predictor of a 2-fold increase in mortality rate in patients with STEMI. However, the CRP/albumin ratio was not useful in determining infarct localization and width. Rather, we recommend the use of the CRP/albumin ratio in conjunction with other prognostic scoring systems or laboratory parameters to predict the short-term prognosis of patients with STEMI, as it is an inexpensive, reproducible, systemic biomarker of inflammation and therefore of CAD. Further randomized and controlled studies with larger sample sizes are needed to confirm these findings.

## Ethical approval and informed consent

This study was conducted in accordance with the 1989 Declaration of Helsinki and was approved by the Ethics Committee of the Faculty of Medicine, University of Health Sciences, İstanbul Research and Training Hospital (ethical approval number 1453). A written informed consent was obtained directly from the patient or from his or her authorized representative.
